# Supplementary material for: Identification of photoperiod-induced specific miRNAs in the adrenal glands of Sunite sheep (Ovis aries)
Source: Front Vet Sci. 2022 Jul 22;9:888207. doi: 10.3389/fvets.2022.888207 (PMC9354845; doi:10.3389/fvets.2022.888207)
Supplement: Supplementary file 1 [file Data_Sheet_1.ZIP › Supplementary file/miRDeep2 file.zip!/miRDeep2 file/Novel_Structure/SP42A3/Novel_221.pdf]

Provisional ID	:	NC_019478.2_27874
Score total	:	1.9
Score for star read(s)	:	-1.3
Score for read counts	:	0
Score for mfe	:	1.6
Score for randfold	:	1.6
Score for cons. seed	:	
Total read count	:	556
Mature read count	:	556
Loop read count	:	0
Star read count	:	0

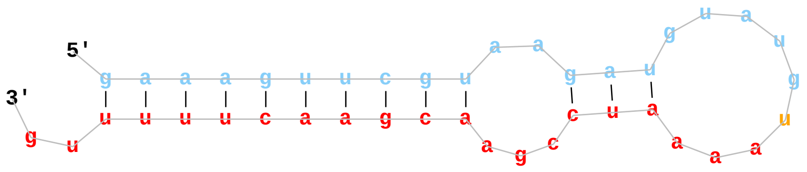

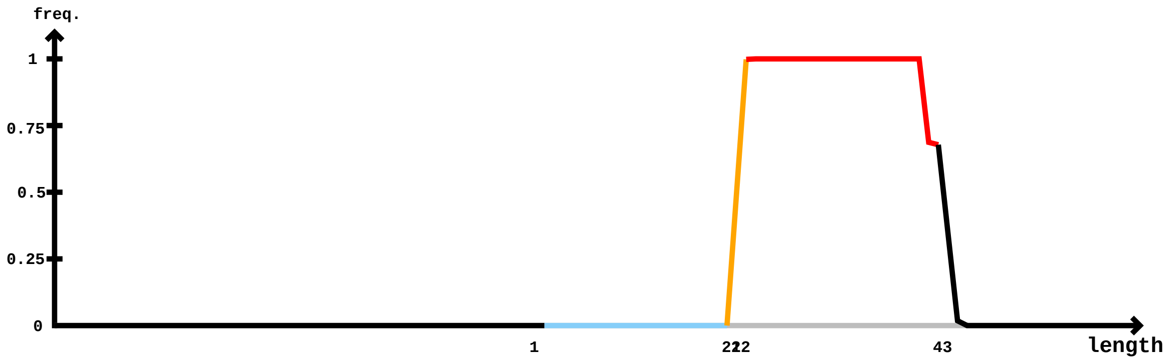

Star Mature

```
5'- gcagaggaaggaaggagggggugucugagaggguauugggcuugggccagaaaguucguaagauguauguaaaauccgaacgaacuuuuugggcccacccaguauuucaaa   -3'    exp
    .((((.(..............).))))...(((((((((..((((((((((((((((..(((.........)))...))))))))))).)))))))))))))))....   reads    mm    sample
    .....................................................................uaaaauccgaacgaacuuuuugg...................   1        0     seq
    ......................................................................aaaauccgaacgaacuuuu......................   173      0     seq
    ......................................................................aaaauccAaacgaacuuuuu.....................   5        1     seq
    ......................................................................aaaauccgaacgaacuuuuug....................   362      0     seq
    ......................................................................aaaauccAaacgaacuuuuug....................   3        1     seq
    ......................................................................aaaauccAaacgaacuuuuugg...................   1        1     seq
    ......................................................................aaaauccgaacgaacuuuuuggg..................   10       0     seq
    .......................................................................aaauccgaacgaacuuuu......................   1        0     seq
```
